# CAIcal: A combined set of tools to assess codon usage adaptation

**DOI:** 10.1186/1745-6150-3-38

**Published:** 2008-09-16

**Authors:** Pere Puigbò, Ignacio G Bravo, Santiago Garcia-Vallve

**Affiliations:** 1 Department of Biochemistry and Biotechnology, Rovira i Virgili University (URV). Campus Sescelades, c/Marcelli Domingo s/n, 43007 Tarragona, Spain; 2 National Center for Biotechnology Information, National Library of Medicine, National Institutes of Health. Bethesda, Maryland 20894, USA; 3 Experimental Molecular Evolution, Institute for Evolution and Biodiversity, University of Muenster, Germany

## Abstract

**Background:**

The Codon Adaptation Index (CAI) was first developed to measure the synonymous codon usage bias for a DNA or RNA sequence. The CAI quantifies the similarity between the synonymous codon usage of a gene and the synonymous codon frequency of a reference set.

**Results:**

We describe here CAIcal, a web-server available at  that includes a complete set of utilities related with the CAI. The server provides useful important features, such as the calculation and graphical representation of the CAI along either an individual sequence or a protein multiple sequence alignment translated to DNA. The automated calculation of CAI and its expected value is also included as one of the CAIcal tools. The software is also free to be downloaded as a standalone application for local use.

**Conclusion:**

The CAIcal server provides a complete set of tools to assess codon usage adaptation and to help in genome annotation.

**Reviewers:**

This article was reviewed by Purificación López-García, Dan Graur, Rob Knight and Shamil Sunyaev.

## Background

Ever since a relatively high number of DNA sequences were publicly available in databases, several statistical analyses addressing DNA composition have been performed. One of the parameters that first interested the scientist was codon usage [1]. It was soon discovered that a considerable heterogeneity in the codon usage exists between genes within species and that the degree of codon bias is positively correlated with gene expression [2,3]. To quantify the degree of bias in the codon usage of genes, several parameters or indices have been worked out. The Codon Adaptation Index (CAI) developed by Sharp and Li [4], rapidly became one of the most used indices. The CAI is a measure of the synonymous codon usage bias for a DNA or RNA sequence and quantifies codon usage similarities between a gene and a reference set. The index ranges from 0 to 1, being 1 if a gene always uses the most frequently used synonymous codons in the reference set. The CAI has been used for estimation of gene expressivity and for prediction of highly expressed genes [5-9]; for giving an approximate indication of the likely success of heterologous gene expression [7]; for detecting dominating synonymous codon usage bias in genomes [3]; for acquiring new knowledge about species lifestyle [3,10]; and for studying cases of horizontally transferred genes [11,12].

## Results and discussion

The most important contribution that we aim to provide with our server is to tie together several features, previously existing but disseminated throughout the Internet, and some new features related to CAI calculation and analysis, and to implement them into a single and easy-to-use web site.

### Description of the CAIcal server

The CAIcal web-server, freely available at , calculates the CAI for a group of sequences using different reference sets and includes a complete set of tools related with codon usage adaptation, e.g. the representation of the CAI along a sequence or multialignment and the estimation of an expected CAI value (eCAI). CAI is calculated following the original method proposed by Sharp and Li [4] but using the recent computer implementation proposed by Xia [13]. In the following subsections we describe the inputs of the server and its main features.

### Inputs of the server

The inputs for the server depend on the calculation to be performed. The basic inputs for calculating CAI are the query sequences, the reference set and the genetic code used for translation. The query sequences must be DNA or RNA sequences in fasta format. The server first checks whether the query sequences are a DNA or RNA region that codifies a protein. The reference set required to calculate the CAI can be introduced in a variety of formats, including that of the Codon Usage Database [14]. A direct link to this database is provided in the CAIcal interface. This database contains codon usage tables extracted from GenBank and organized by species. Several of the calculations available in CAIcal, such as the CAI calculation and its representation in a sequence, can be used with two reference sets simultaneously. Therefore, it is easier to compare the codon usage of a gene with respect to the codon usage of two different organisms and check whether it is more adapted to one of them. See the tutorial available from the server home page for a complete description of errors and warnings and for more information about input requirements.

### Set of tools

A number of programs and servers that calculate CAI for a gene or a group of genes are available elsewhere, such as CodonW, EMBOSS [15], CAIJava [3], CAI Analyser [8], as well as JCAT [16] and the CAI Calculator [5]. All of these tools represent valuable resources.

The server first provides a number of basic calculations that are also available elsewhere:

(i) The absolute and synonymous codon usage of a group of DNA sequences and other useful parameters such as length, total G+C content and G+C content at the three codon positions, and the effective number of codons [17].

(ii) The CAI of a DNA sequence or group of sequences. This index measures the adaptation of the synonymous codon usage of a gene to the synonymous codon usage of up to two reference sets that can be chosen by the user.

The new features incorporated in this server are:

(iii) An expected value of CAI [18] is determined by randomly generating 500 sequences from the G+C content and the amino acid composition of the query sequences. This expected CAI therefore provides a direct threshold value for discerning whether the differences in the CAI value are statistically significant and arise from the codon preferences or whether they are merely artefacts that arise from internal biases in the G+C composition and/or amino acid composition of the query sequences. The E-CAI module that calculates the expected CAI values has been previously described [18]. Additionally, one of the tools included in CAIcal is a graphical local user interface that can be downloaded and allows the calculation of the CAI and eCAI of hundreds or thousands of sequences on a whole-genome scale easily [18].

(iv) The weight of each codon, i.e. the frequency of codon use compared to the frequency of use of the optimal codon for that amino acid in the reference set, can be graphically represented along a DNA sequence using a sliding window defined by the user. This result provides an intuitive visualisation of the changes in the CAI throughout the input and identifies discontinuities that might correlate with informational and/or operational features of the DNA sequence. The CAIscan tool of the CAI Analyser package [8] allows a similar analysis, i.e. scanning a sequence calculating the CAI over a selected window.

(v) A graphical representation can be made of the weight of each codon along a multiple protein alignment that has been translated to a DNA alignment using a unique reference set for all the sequences of the alignment or using a reference set for each sequence. The inputs for this option are a protein multialignment in clustal format, the DNA sequence of each of the sequences of the multialignment with the same identification field between the DNA and protein sequences and one or more codon usage tables to use as reference sets. This result provides a graphical display that enables the protein sequence alignment to be correlated with the informational/compositional content of the DNA sequence that encodes them.

**Figure 1:**
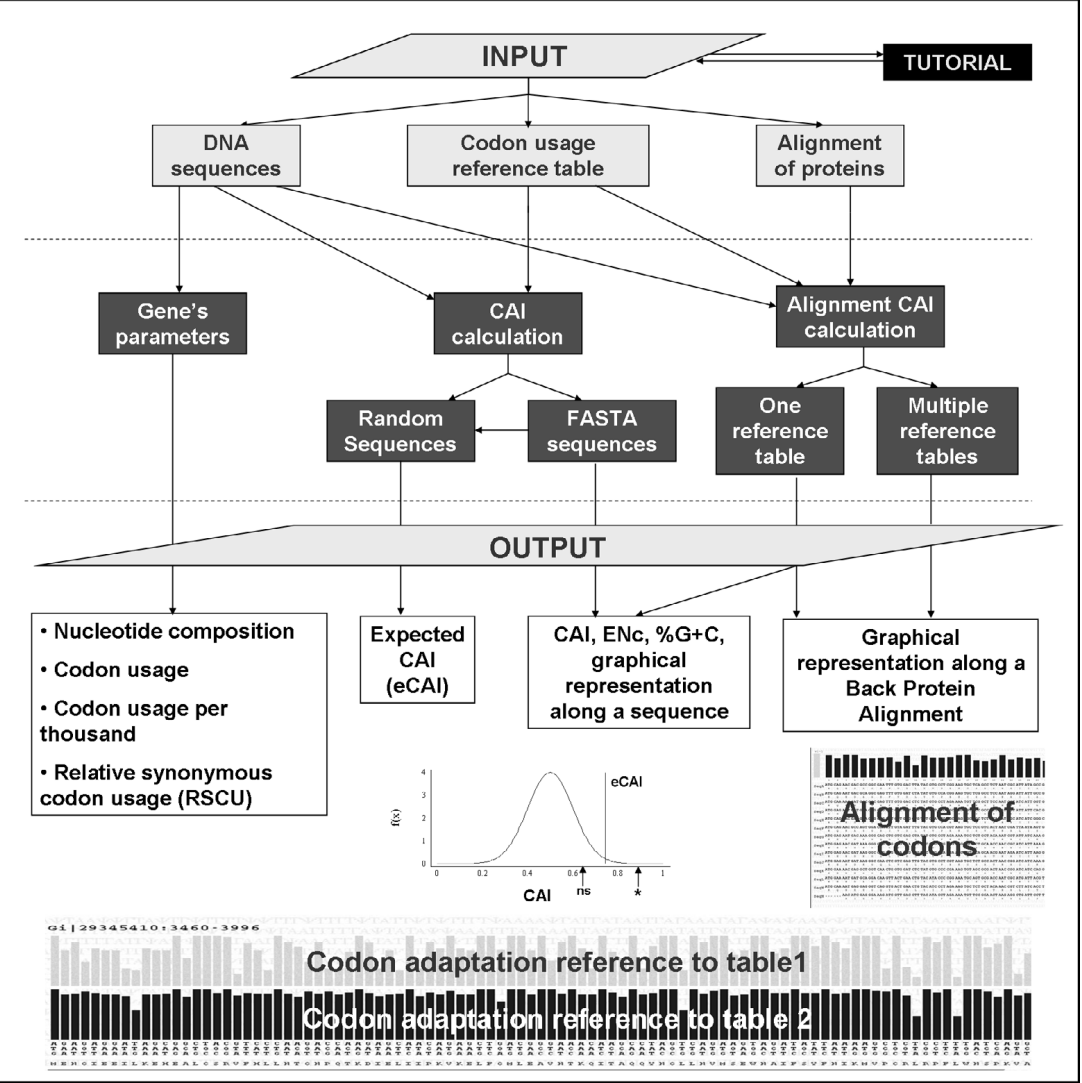
**Schematic representation of the options available in the CAIcal server**. Using a combination of three inputs (DNA or RNA sequences, a codon usage reference table and/or a protein alignment), the server calculates gene parameters such as %G+C, Relative Synonymous Codon Usage (RSCU) and Effective Number of Codons (ENc), the CAI for one or more DNA or RNA sequences and an expected CAI and represents the CAI along a DNA sequence or in a protein multialignment translated to DNA.

The options available in the server are summarized in Figure 1. All these options are accessible from the main page of the server and several links have been created between them. As an example, after the CAI value of a group of sequences has been calculated, an expected CAI value can be estimated or the graphical representation of the CAI value along each sequence can be visualized. Several parameters used in the calculations, such as the window length in the graphical representation of the CAI along a sequence or the upper confidence limit to estimate an expected CAI, are defined by the user. The results are therefore flexible and fit the needs of the user. For the results, the server produces several tables and graphs together with several text boxes containing the results in a tab-delimited format have been created, which makes it easy to copy and paste them into spreadsheet programs. Finally, a tutorial, a Frequently Asked Questions (FAQ) section and several examples are available from the home page of the server.

### Example of how to use the CAIcal server

**Figure 2:**
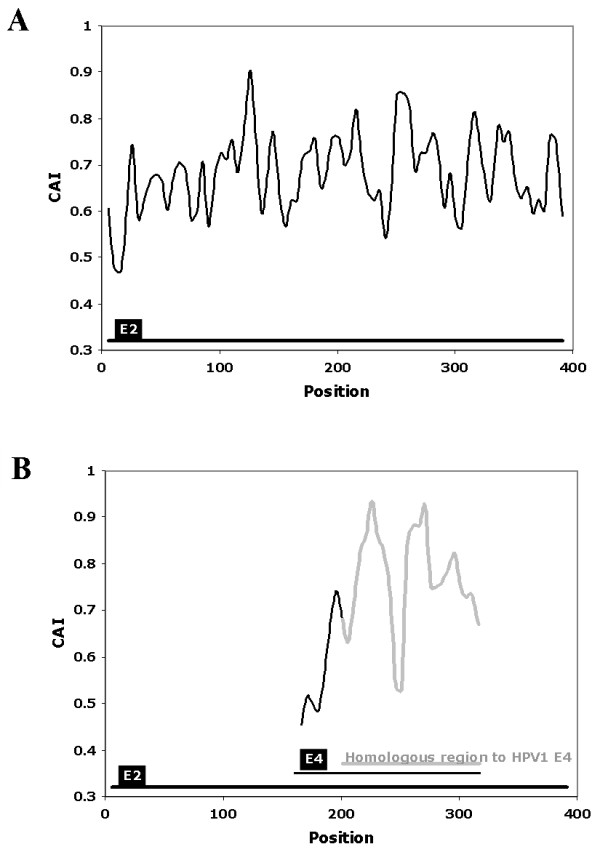
**Representation of the CAI, calculated using the human mean codon usage as a reference set, in the DNA sequence that encodes HPV63 E2 and E4**. Part A represents the reading frame that encodes E2. Part B represents the same HPV63 genome fragment that encodes E2, but in the reading frame +1, which contains E4. The grey line in B represents the fragment of HPV63 E4 homologous to the closely related HPV1 E4. The black line in B represents the stretch also annotated as HPV63 E4, but which lacks homology with HPV1 E4. Note that the initial E4 region from HPV63, which is not homologous to the HPV1 gene, has an extremely low CAI, which suggests a wrong annotation for the E4 gene in HPV63. This figure was obtained using the output of the calculation of CAI along a sequence of the CAIcal server, with a window length of 11 and a window step of 5.

The CAIcal was used to annotate the genomic discontinuity in the E4 gene of human papilomavirus 1 (HPV1). Papillomaviruses (PVs) are a family of small dsDNA viruses that cause a variety of diseases including cervical cancer. The genome of PVs is modular with three different regions, each of which has a different evolutionary rate [19,20]. These regions are: an upstream regulatory region, an early region that codes for proteins (e.g. E1, E2, E4, E5, E6 and E7) involved in viral transcription, replication, cell proliferation and other steps of the viral life cycle, and a structural region that contains two genes that code for the capsid proteins L1 and L2. A general characteristic of genes encoded in human PVs is their peculiar codon usage preference compared to the preferred codon usage in human genes [21,22], although the exact reason for this poor adaptation to the genome of their host is still unknown. Like other viral genomes, some of the PV genes overlap partially or completely. This is the case of the E4 gene, which is completely nested within the E2 gene in a different reading frame [23]. The function of E4 is not completely understood and its annotation is not very rigorous [14]. The mature E4 protein appears after splicing, with the donor site situated some codons downstream from the start codon of the E1 gene, and the acceptor site situated close to the middle of the E2 gene [24,25]. The fact that most of E4 overlaps with E2, that the mature E1^E4 protein contains a few amino acids from E1 and that the splice sites are not strictly conserved, makes it difficult to determine the true E4 sequence *in silico*. The E4 PVs genes available in the databases are therefore very different in length and similarity. Although the genomes of many PVs have been sequenced, information about the expression of their genes or cDNA sequences is only available for a few of them. One of these is HPV1. In this case, the annotation of the HPV1 E4 gene is confirmed by mRNA data [26]. However, the E4 gene from HPV63, a PV that is phylogenetically related to HPV1 [19,20,27], is longer than the E4 gene from HPV1. The difference is between both sequences is 96 nucleotides located at 5' end of HPV63 E4. We can use the CAIcal server to show that the codon usage of these 96 nucleotides at the beginning of HPV63 E4 is very different from that of the rest of the E4 sequence, measured as the CAI value calculated with the human codon usage as reference (figure 2). This suggests that the acceptor splice site of HPV63 E4 is not well annotated and that the true E4 nested within E2 probably starts downstream from the annotated position.

## Conclusion

The CAIcal server provides a complete set of tools to assess codon usage adaptation and helps to annotate genomic discontinuities such as the donor splicing site of the E4 ORF of papilomaviruses.

## Competing interests

The authors declare that they have no competing interests.

## Authors' contributions

PP designed the server, made the programming task and drafted the manuscript. IGB participated in design of the server, prepared the example, and helped draft the manuscript. SG-V conceived and designed the server, coordinated the project and drafted the manuscript. All authors read and approved the final manuscript.

## Reviewers' comments

### Reviewer's report 1: Purificación López-García, CNRS, Université Paris-Sud

This article describes a series of tools for the automatic calculation of the codon adaptation index (CAI) and related measurements from input and reference data that have been implemented in a web-based server . CAI values are useful for a variety of purposes going from genomic annotation and gene expression analyses to the detection of potential horizontal gene transfer events. Although, as pointed out by the authors, a number of freely available facilities providing the calculation of CAI exist already, this new set of tools offers the possibility to obtain some additional estimates. These include the calculation of expected CAIs from randomly generated sequences with the GC content and amino acid composition of the input sequences that can be compared then with the observed CAIs, as well as measurements of the weight of each codon and their graphical representation. An example of the possible utility of these CAI measurements to test and validate annotations is provided. I find that this group of tools accessible online will be useful to the scientific community. I hope that this web-based server will benefit and get improved with the progressive input and suggestions of a wide variety of users.

### Reviewer's report 2: Dan Graur, Department of Biology and Biochemistry, University of Houston

A very simple and straightforward tool for dealing with codon usage. I have no other comments.

### Reviewer's report 3: Rob Knight, University of Colorado

In this manuscript, Puigbo *et al*. describe their CAIcal web server. CAI, the Codon Adaptation Index, is an important concept relating codon usage to gene expression. Although several software tools online already calculate CAI, CAIcal appears to offer a unique combination of functionality that is not easily duplicated using other tools.

However, the tool in its current form would appear to be a relatively minor advance over existing tools, and I would strongly encourage the authors to consider an extensive overhaul of the software and the manuscript before publication. However, I think the present work contains the seeds of a useful contribution to the field and to the literature, and definitely encourage the authors to persevere, perhaps thinking more carefully about the target audience of the software and the paper.

More attention needs to be paid to the specific contribution of this work if it is to be published as an independent piece of software. No feature of this tool really appears to be unique, e.g. the plots of CAI along a gene and codon-by-codon are also in Codon Analyser (as the authors note), many tools allow calculation of CAI against a reference set, etc.

**Authors' response**: *As we acknowledge in the manuscript, a number of tools are available elsewhere addressing different calculations around CAI. We consider however, that one of the strengths of the CAIcal server is to gather together pre-existing and new features into a single and easy-to-use web site, as you also note in your revision "CAIcal appears to offer a unique combination of functionality that is not easily duplicated using other tools". As an example, after the CAI value of a group of sequences has been calculated, the user can easily (with only a click of the mouse) estimate an expected CAI value for discerning whether the differences in CAI are statistically significant or whether they are merely artifacts. The graphical representation of the CAI value along each sequence can also be easily visualised. In addition, we also want to point out the usability of the server, used to denote here the ease with which people can employ a particular tool. Thus, several of the existing tools that allow calculation of CAI are not web-servers; other require some kind of installation or execution; and some of them provide easy calculations that lack in flexibility. Finally, the server allows to represent the CAI value along a protein multialignment back-translated to DNA, a feature currently not available elsewhere*.

Similarly, the calculations of the expected CAI values are delegated to another tool, E-CAI, that the authors have previously published, but this is not very clear from the description in the paper. If the sole contribution is to tie together several pre-existing features into a single web site, the authors need to make the case much more clearly that this combination will be of use to end users in a way that the individual pre-existing tools are not.

**Authors' response**: *We have added a new sentence in the paper clarifying this point*.

I think the source code of the standalone version needs a substantial overhaul before publication. It is full of large, error-prone tables of redundant information about genetic codes, for example, which should be dynamically calculated from a compact, standardized and easily verified source (e.g. the NCBI genetic code tables), is essentially without useful comments, mixes presentation and logic, and has many other indicators of poor coding style (for example, it looks as though several separate applications have simply been pasted together).

**Authors' response**: *Although the main aim of our work was to provide a web-based server for CAI analysis, this was a fair criticism. The source code needed an extensive revision of style and lacked useful comments that could guide the experienced user. We have largely rewritten it and it incorporates now numerous comments about the functionality of each different part. Thus, we have developed the local version 1.3. The source code in the standalone application follows a descendent algorithm rather than several separate applications have simply been pasted together. For the sake of clarity, we have included a file with a detailed description of the CAIcal functions (this file is available from the web site in the FAQs section *– . *The standalone application includes now new functions related with genetic codes to avoid putative error-prone in tables. Though, again, you are right and the coding style could still be improved, the program works well*.

Although I appreciate that the authors have made the effort to produce and distribute a standalone version, the code unfortunately does not inspire confidence in the web site either in this case. Test cases, e.g. using Perl's built-in unit testing framework, would definitely be a useful addition to verify that the calculations are correct.

**Authors' response**: *This was an interesting suggestion that we have addressed. To verify that the calculations are correct, we show that the results of the two independent programs (the standalone version written in Perl and the web-server written in PHP) are the same. In addition, we have compared our results with the results using other existing programs and the results are not significantly different. A file with some tests we made is available from the web site in the FAQs section *.

The utility of the Monte Carlo approach is also somewhat unclear to me, as it appears that the expected CAI could be calculated analytically, along with confidence intervals, using the multinomial distribution. It is possible that this is not feasible for numerical reasons, but some justification of the approach would be useful.

**Authors' response**: *The expected CAI is calculated analytically from the CAI values of 500 randomly generated sequences with the same G+C content and amino acid composition as the query sequences. However, the Monte Carlo approach is used to generate the random sequences, not to calculate the expected CAI. In this sense, please see also Question 15 at the FAQs section of the server *.

I did not find the example especially compelling, but this is a relatively minor criticism and I understand that it is likely that the authors would want to publish any especially interesting results separately from the description of the tool itself. However, it might be interesting to try to reproduce a well-known conclusion from existing work to show how much easier it is with this workflow than with pre-existing tools. There are many examples in the literature as CAI is such a widely-used technique.

The manuscript and the web site need substantial attention to the quality of the English. I have not corrected minor wording and grammatical errors in this version of the manuscript, but if the authors plan to publish this manuscript regardless of the above comments, I would definitely recommend careful attention to detail, and also removing formatting errors such as the text "Sub-heading for this section" on page 3. Overall, I think this is a good first attempt and could ultimately be revised into a useful contribution that is more suitable for publication.

**Authors' response**: *After receiving your comments and the comments of the three additional referees, we have decided to rewrite the code, to revise the manuscript and to publish it. We would like to thank you again for your comments. We think that it is not necessary any further overhaul of the software, as we agree that some changes were necessary in the manuscript and in the source code of the standalone version, and have accordingly been performed. We are glad to acknowledge that the code is easier to read after introducing the comments you suggested. Additional changes in the manuscript include also a second revision of the quality of the English following the recommendations by the NIH Fellows Editorial Board, and some clarifications. We sincerely consider that we have addressed the criticism you raised to the previous version of the manuscript*.

### Reviewer's report 3 (second revision): Rob Knight, University of Colorado

The revised versions of the manuscript and software are significantly improved.

### Reviewer's report 4: Shamil Sunyaev, Harvard Medical School

This manuscript presents a new online tool to compute codon adaptation index (CAI). Although there are several CAI calculators available online, this new server includes several additional features such as computation of expected CAI and visualization of changes in the CAI along the sequence. The authors also present an analysis of papilomavirus as an example of the server utility. In sum, the manuscript does not report any significant novel scientific findings but presents a tool potentially useful for the research community.
